# A Mendelian randomization study reveals a causal association between NASH and the risk of atrial fibrillation

**DOI:** 10.3389/fendo.2025.1390259

**Published:** 2025-03-18

**Authors:** Biwei Cheng, Xuekang Su, Jue He, Yanghui Gu, Mingtai Chen, Yi Wei, Yumeng Yi, Peiying Chen, Xiaojuan Lin, Tao Li, Chong Xu, Qiang Liu, Biao Li

**Affiliations:** ^1^ Shenzhen Traditional Chinese Medicine Hospital, Shenzhen, Guangdong, China; ^2^ The Fourth Clinical Medical College, Guangzhou University of Chinese Medicine, Shenzhen, China

**Keywords:** atrial fibrillation, non-alcoholic fatty liver disease, non-alcoholic steatohepatitis, Mendelian randomization, causal relationship

## Abstract

**Background:**

Epidemiological evidence suggests that non-alcoholic fatty liver disease (NAFLD) may increase the risk of atrial fibrillation (AF). However, the findings are inconsistent, and the causality remains to be established.

**Methods:**

We conducted two-step, two-sample Mendelian randomization (MR) analysis to assess the association between genetically predicted NAFLD (i.e. chronically elevated serum alanine aminotransferase levels [cALT], imaging-based and biopsy-confirmed NAFLD) and AF. Subsequently, we further performed Mendelian randomization to investigate the causal relationship between non-alcoholic steatohepatitis (NASH), a subtype of NAFLD, and AF. The inverse variance weighted (IVW) method was used as the primary approach to reveal the potential causation between the exposure and outcome.

**Results:**

There was no significant causal association between NAFLD diagnosed based on cALT, confirmed by imaging, or verified by biopsy, and an increased risk of atrial fibrillation. Furthermore, the results of the IVW method revealed a positive causal effect of NASH on AF (OR=1.113, 95% CI=1.025-1.209, P = 0.011). In the reverse analysis, however, no evidence supported a significant genetic association between AF and NASH (OR=0.974, 95% CI=0.934-1.016, P = 0.214).

**Conclusion:**

A causal relationship existed between NASH and the risk of AF. However, no significant genetic association has been observed between NAFLD and AF risk. This suggests that managing the progression of NAFLD may hold potential value in preventing the onset of AF.

## 1 Introduction

Non-alcoholic fatty liver disease (NAFLD) is a chronic metabolic liver disease, primarily encompassing simple steatosis and non-alcoholic steatohepatitis (NASH) (1). Simple steatosis is defined as the presence of ≥5% hepatic steatosis (HS) without evidence of hepatocellular injury in the form of hepatocyte ballooning. NASH is defined as the presence of ≥5% HS and inflammation with hepatocyte injury (e.g., ballooning), with or without any fibrosis. NASH represents a more severe pathological manifestation compared to steatosis alone and poses a heightened clinical risk of progressing to liver fibrosis, cirrhosis, and ultimately, hepatocellular carcinoma (2). Meanwhile, a growing body of evidence demonstrates that NAFLD, as a multisystemic disease, affects extrahepatic organs and regulatory mechanisms, thereby elevating the risk of cardiovascular diseases, type 2 diabetes, and other related conditions (3). Recently, the concept of metabolic association with fatty liver disease (MAFLD) has been proposed to better reflect the metabolic basis of the disease, emphasizing its association with obesity, insulin resistance, and cardiovascular risk (4).

As a cardiovascular disease with rapidly rising incidence and prevalence, atrial fibrillation (AF) significantly increases the global disease burden (5). Studies have shown that NAFLD is closely associated with the development of AF (6–8). NAFLD and AF share common risk factors, including obesity, insulin resistance, type 2 diabetes, systemic inflammation (9), and abnormal circadian rhythms (10–12), which can contribute to the development of both diseases. Additionally, the pro-inflammatory and pro-fibrotic states during the progression of NAFLD may contribute to atrial remodeling, thereby increasing the risk of atrial fibrillation (13). However, whether NAFLD is an independent risk factor for AF remains controversial.

Mendelian randomization (MR), a research method used to infer causal relationships between exposures and outcomes from a genetic perspective, is currently being widely applied in medical research (14). To further explore whether NAFLD is an independent risk factor for AF, our study employed the two-sample Mendelian randomization (TSMR) to clarify the potential causal relationships between NAFLD, particularly its advanced phenotype (NASH), and AF.

## 2 Methods

### 2.1 Study design

**Figure 1:**
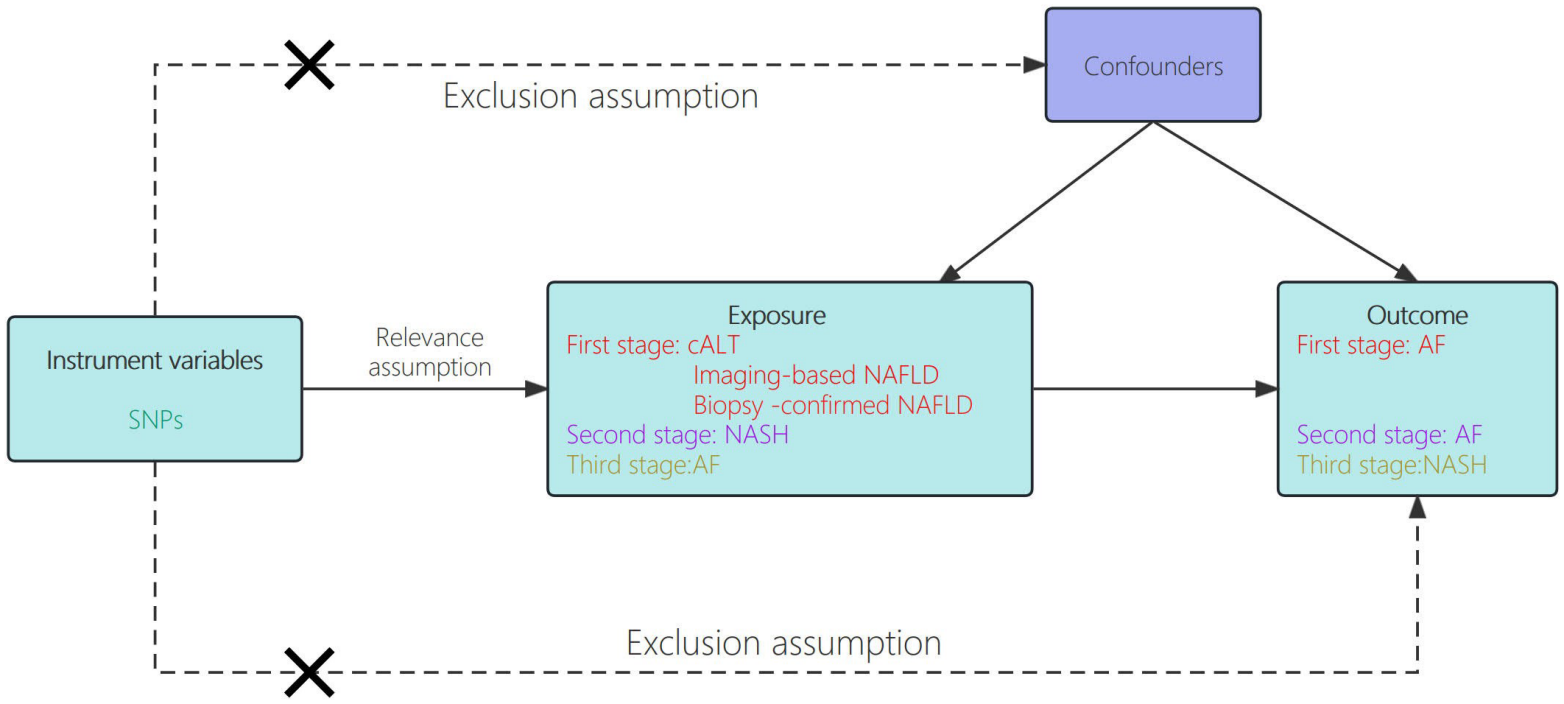
Schematic showing how Mendelian randomization was used to evaluate a causal association between NAFLD/NASH and AF in this study.

The genetic data pertaining to exposure and outcome variables in this study were derived from summary-level data of genome-wide association studies (GWAS). We performed TSMR analysis with publicly available summary‐level data, which were derived from several large‐scale cohorts (15–17). Declaration of Helsinki statement and informed consent procedure has been described in the original publications of these cohorts. In the first phase, we conducted two-sample Mendelian randomization analysis to investigate the causal relationship between NAFLD and AF. In the second phase, we employed the same two-sample Mendelian randomization approach to examine the causality between NASH (advanced phenotype of NAFLD) and AF. The flowchart of the study design is shown in **Figure 1**.

### 2.2 Data sources

Genetic data relating to NAFLD were derived from a recently published GWAS for cALT, in which NAFLD was defined as an elevated alanine transaminase (ALT) > 40 U/L for men or >30U/L for women during at least two time points at least 6 months apart within a 2‐year period, after exclusion of other liver diseases (15). The study included 90,408 cases of cALT and 128,187 controls from the Million Veteran Program (MVP) database, predominantly composed of European Americans (EA, 75.1%) across four ancestral groups. Overall, 77 independent single-nucleotide polymorphisms (SNPs) were identified with significant associations (P < 5e-8) in the study. Among the SNPs, 22 were replicated in a subsequent imaging-confirmed NAFLD cohort (n=44,289), while 36 were replicated in a biopsy-defined NAFLD cohort comprising 7,397 cases and 56,785 controls. Therefore, NAFLD defined based on cALT, imaging, and biopsy was separately included in the MR analysis.

The SNPs associated with NASH are sourced from the FinnGen R10 dataset within the FinnGen study (16). The study is a large-scale genomics initiative that has analyzed over 500,000 Finnish biobank samples and correlated genetic variation with health data to understand disease mechanisms and predispositions. The project is a collaboration between research organisations and biobanks within Finland and international industry partners. The definition of NASH refers to clinically diagnosed non-alcoholic steatohepatitis (ICD-10 code K73.80). The study included a total of 175 NASH cases and 412,006 control subjects.

Genetic data associated with AF were extracted from the largest GWAS meta-analysis of 60,620 AF cases and 970,216 controls of European ancestry (17). This comprehensive dataset integrated information from notable studies such as the Nord-Trøndelag Health Study (HUNT), deCODE, the Michigan Genomics Initiative (MGl), DiscovEHR, UK Biobank, and the AFGen Consortium. AF cases were defined by clinically diagnosed atrial fibrillation or flutter (ICD-10 code I48 and ICD-9 code 427.3). A detailed description of the GWAS data involved in this study is shown in **Table 1**.

**Table 1:** Detailed description table of GWAS data involved in this study.

Phenotype	PMID or GWAS ID	Years	Population	Sizes of sample	Consortium or cohort study
cALT	35654975	2022	European‐American, African‐American, Hispanic‐American, and Asian‐American	90,408 cases and 128,187 controls	–
Imaging‐based NAFLD	35654975	2022	European‐American, African‐American, and Hispanic American	44,289	–
Biopsy-confirmed NAFLD	35654975	2022	European‐American and Hispanic American	7,397 cases and 56,785 controls	–
NASH	–	2023	European	175 cases and 412,006 controls	FinnGen
AF	30061737	2018	European	60,620 cases and 970,216 controls	HUNT, deCODE, MGI, DiscovEHR, UK Biobank, and AFGen Consortium

### 2.3 Screening for genetic instrumental variables

All instrumental variables included into the ultimate MR analysis are required to fulfill three fundamental assumptions. Firstly, SNPs that are significantly associated with exposure on a genome-wide scale are considered (specifically, p < 5e-8 for cALT, p < 5e-6 for Imaging-based NAFLD, Biopsy-confirmed NAFLD, and NASH, with 1.44e-9 representing 0.05/nSNPs for AF). Secondly, SNPs without linkage disequilibrium (kb = 10000, r² < 0.001) are extracted, and palindromic SNPs with intermediate allele frequencies are excluded. Proxy SNPs were not used in this MR analysis. The results from IVW will serve as the main outcomes for TSMR analysis. Significant associations identified by IVW will undergo further sensitivity analysis, and pleiotropy will be tested using MR-Egger to ensure the effects of horizontal pleiotropy. The strength of the selected genetic instrument was assessed using F statistics, with a mean F‐statistic < 10 regarded as a weak set of instrumental variables.

## 3 Results

### 3.1 Causal effects of NAFLD-related traits on AF

**Figure 2:**
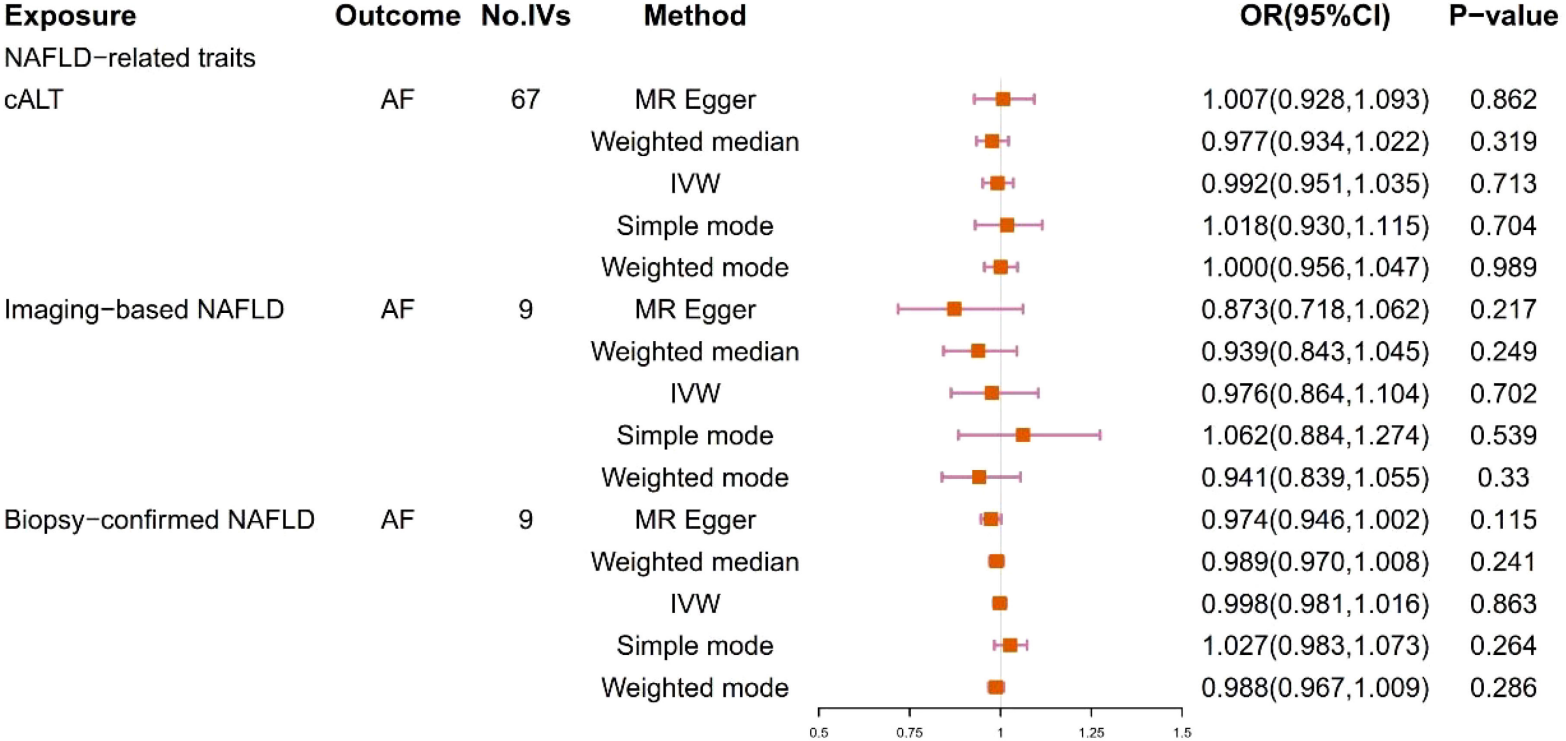
Odds ratios and p-value of MR analysis for the associations between NAFLD and AF. OR, odds ratio; 95% CI, 95% confidence interval.

**Figure 3:**
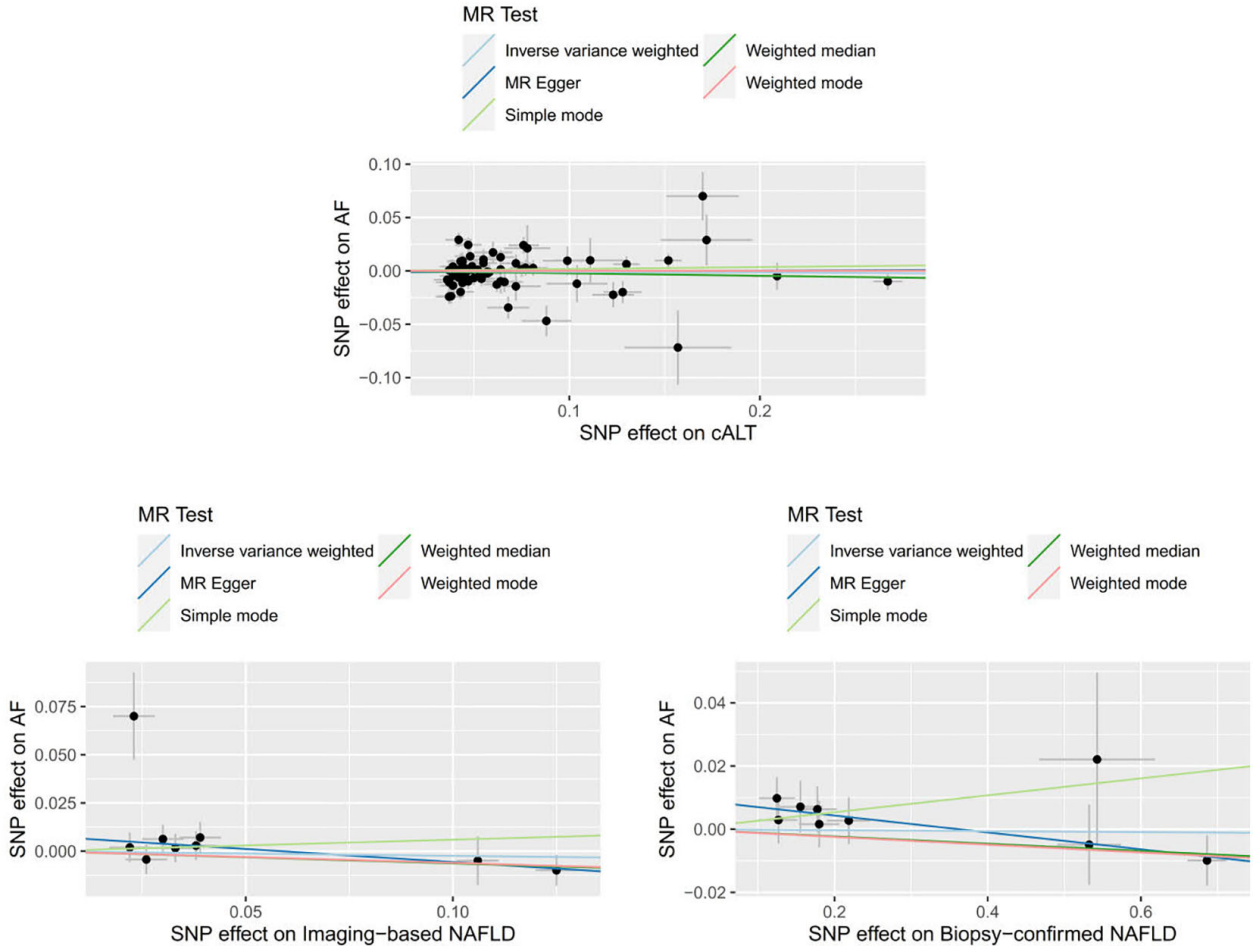
Scatter plots to assess causal associations between NAFLD-related traits and AF.

We obtained 67 cALT‐associated SNPs, 9 imaging‐associated SNPs and 9 biopsy‐associated SNPs after removing correlated SNPs (**Supplementary Tables 1**-**3**). According to the IVW result, no significant causal relationship between NAFLD-related traits and the risk of AF was found. Furthermore, other MR methods showed consistent results. **Figures 2** and **3** present the detailed results of the MR analysis.

### 3.2 Causal effects between NASH and AF

#### 3.2.1 The effect of NASH on AF

**Figure 4:**
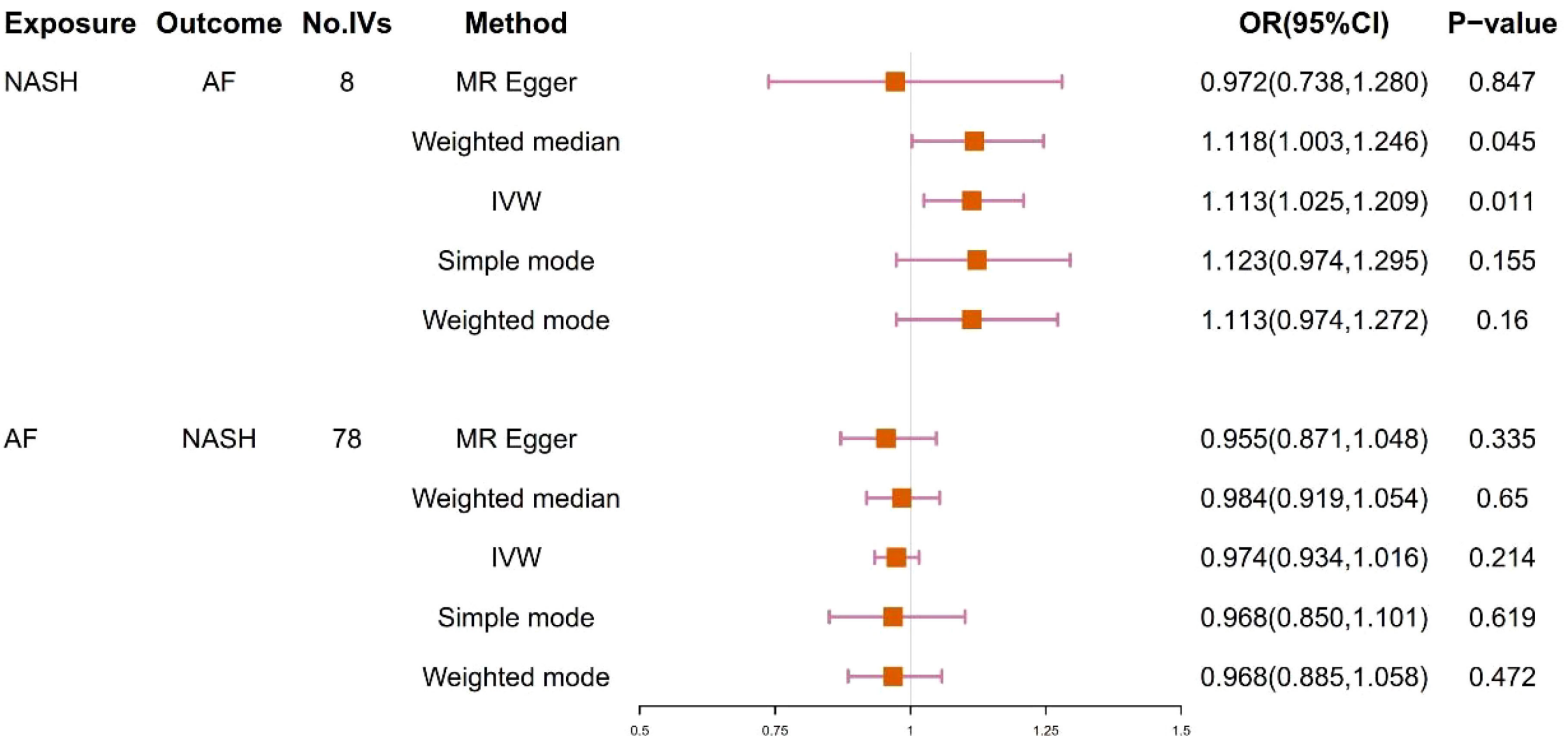
Odds ratios and p-value of MR analysis for the associations between NASH and AF. OR, odds ratio; 95% CI, 95% confidence interval.

**Figure 5:**
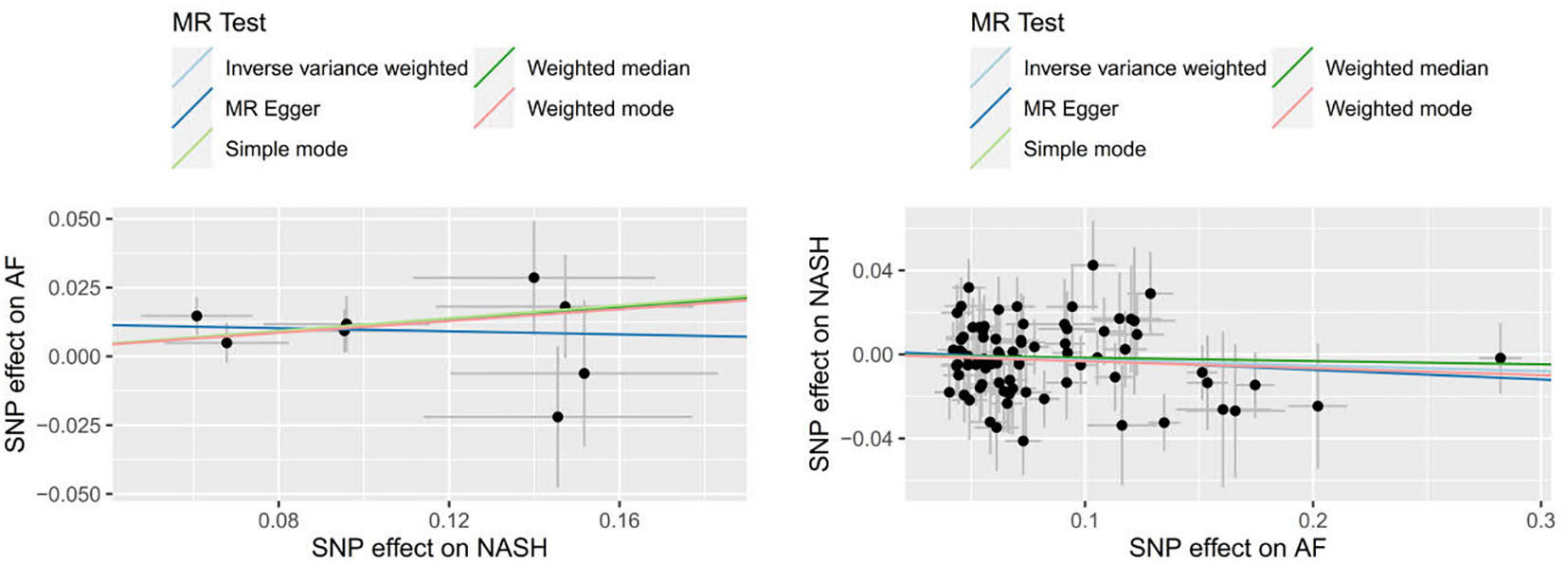
Scatter plots to assess causal associations between NASH and AF.

9 SNPs based on clinical diagnosis of NASH were used in MR analysis (**Supplementary Table 4**). IVW method showed a statistically significant association between genetically predicted NASH and the risk of AF (OR=1.113, 95% CI=1.025-1.209, P = 0.011). The result of Weighted median was consistent with IVW (p=0.045), while the remaining three methods were not statistically significant. **Figures 4** and **5** present the detailed results of the MR analysis.

Cochran’s Q statistic showed no significant heterogeneity in the estimates of included SNPs (Q=4.883, P=0.674). The intercept of MR-Egger was 0.013 (P = 0.350), which showed no significant horizontal pleiotropy. In addition, the MR-PRESSO Test also showed that there was no horizontal pleiotropy in this study (Global Test P= 0.769), and no potential outliers were found in the result, indicating that the result was robust and reliable (**Supplementary Table 6**).

#### 3.2.2 The effect of AF on NASH

78 SNPs depending on the clinical diagnosis of AF were used in MR analysis (**Supplementary Table 5**). There was no significant causal relationship between AF and the risk of NASH according to the result of IVW (OR = 0.974, 95%CI = 0.934–1.016, P = 0.214). In addition, the results of MR Egger, Weighted median, Simple mode and Weighted mode were consistent with IVW. **Figures 4** and **5** present the detailed results of the MR analysis.

## 4 Discussion

Although genetically predicted NAFLD based on cALT diagnosis, imaging and biopsy confirmation was not substantially related to an increased risk of AF, further analysis revealed that NASH, the advanced phenotype of NAFLD, was associated with the risk of developing AF.

### 4.1 The relationship between NAFLD/NASH and atrial fibrillation

Previous clinical studies have shown that NAFLD is closely related to the occurrence of AF. Käräjämäki et al. (18) reported an elevated incidence of AF in NAFLD patients from the OPERA study, which persisted after adjusting for confounding factors. Roh et al. (7) found an independent link between NAFLD (defined by FLI) and increased AF risk in a healthy Korean cohort. Meta-analyses further confirmed a strong correlation between NAFLD and higher AF risk (6, 19–22). As an advanced phenotype of NAFLD, NASH have risk factors for the development of cardiac abnormalities. Whitsett et al. (23) pointed out that AF is highly prevalent in patients with biopsy-proven Nonalcoholic Steatohepatitis (NASH). However, the Framingham Heart Study showed no association between AF and either computerized tomography or ultrasound-diagnosed hepatic steatosis (24).

Mendelian randomization explored the relationship between NAFLD/NASH and AF from the perspective of genetics. Two previous Mendelian randomization studies have explored the relationship between NAFLD and its subtype NASH and AF (Their exposure data originated from the same GWAS study). Their results showed that there was no significant causal relationship between NAFLD and AF, which is consistent with our Mendelian randomization study of NAFLD and AF. Simultaneously, these two investigations probed the causative association between NASH and AF, and the findings indicated the absence of a causal relationship (25, 26). We conducted a Mendelian randomization analysis based on the newly published FinnGen R10 database to further explore the causal relationship between NASH and AF, and the result revealed that genetically predicted NASH was causally associated with an increased risk of AF(OR=1.113, 95% CI=1.025-1.209, P = 0.011). Subsequently, heterogeneity tests and pleiotropy evaluations suggested the causal relationship was robust and reliable(both P > 0.05). Although the increase in odds ratio may not appear substantial, it still holds important epidemiological and clinical implications. Furthermore, reverse MR analysis suggested that AF was not associated with an increased risk of NASH. The larger sample size and updated genetic information in FinnGen R10 may provide a more robust interpretation of the relationship between NASH and AF.

### 4.2 Possible mechanisms associated with NASH and atrial fibrillation

The mechanism of AF induced by NAFLD remains unclear, but it is suggested that chronic inflammation, insulin resistance, lipid deposition, and oxidative stress may be involved in the development of AF in NAFLD patients (27). Mild systemic inflammation is a key factor in NAFLD (28). The Pro-inflammatory environment and oxidative stress lead to an increased release of inflammatory factors, ultimately resulting in an elevated risk of AF (29, 30). Furthermore, as NAFLD progresses to the stage of NASH, lipid deposition leads to the occurrence of lipotoxicity, causing further release of inflammatory factors and exacerbating the inflammatory response (31). During the NASH stage, the degree of liver fibrosis progresses significantly compared to the early stage of NAFLD (simple steatosis). Study have shown that the severity of liver fibrosis in NAFLD patients is associated with an increased risk of atrial fibrillation (AF), potentially mediated through mechanisms such as systemic inflammation, metabolic disturbances, and atrial remodeling (32). Furthermore, Hui et al. reported that high TNF-α levels and hypoadiponectinemia are IR-independent features of NASH, and these two factors synergistically exacerbate insulin resistance, oxidative stress, and lipotoxicity, potentially serving as underlying mechanisms linking NASH to AF risk (33).

### 4.3 The role of NASH in the prevention and treatment of atrial fibrillation

The pathogenesis of atrial fibrillation is still not fully understood, and effective treatment strategies remain limited. Our study provides genetic evidence supporting a causal relationship between NASH and AF risk, which may have implications for clinical practice. When NASH is recognized as a risk factor for atrial fibrillation, patients with NASH, especially those with obesity or metabolic syndrome, should be encouraged to undergo regular screening for atrial fibrillation. In addition, interventions for NASH, such as lifestyle changes (such as weight loss, dietary changes) and anti-inflammatory or anti-fibrotic treatments, may help reduce the risk of atrial fibrillation.

## 5 Conclusion

In conclusion, this MR study reveals a causal relationship between genetically determined NASH and an elevated risk of AF, while there is no apparent causal relationship between NAFLD and AF. This suggests that controlling the further progression of NAFLD may hold potential value in preventing the occurrence of AF.

## 6 Limitation

Although our study had enough statistical power to evaluate the causal relationships, the findings should be interpreted with prudence. Furthermore, this study also has some limitations. Firstly, the selected instrumental variables (IVs) capture only a relatively small proportion of the phenotypic genetic variation, potentially leading to bias from weak IVs. Additionally, the standard for the independence test P-value is relatively lenient (P < 5e-6), which could introduce some bias into the results. Secondly, the definition of NAFLD is partially based on cALT levels rather than the presence of NAFLD itself. Thirdly, a possible limitation of the study is the lack of further stratification for the severity of NAFLD and other factors such as gender and age. The absence of subgroup data for NAFLD limits the extension of these stratified analyses. While our study did not stratify by gender due to data limitations, future research should explore gender-specific associations, particularly in postmenopausal women, to better understand the underlying mechanisms. Finally, our focus was primarily on individuals of European descent, which may reduce the generalizability of our study results.

## Data Availability

The original contributions presented in the study are included in the article/**Supplementary Material**. Further inquiries can be directed to the corresponding author.
